# Bone regeneration in diabetic patients. A systematic review

**DOI:** 10.4317/medoral.22889

**Published:** 2019-06-28

**Authors:** Helena Sábado-Bundó, Mª Ángeles Sánchez-Garcés, Cosme Gay-Escoda

**Affiliations:** 1 DDS. Faculty of Medicine and Health Sciences, University of Barcelona, Barcelona (Spain); 2 MD, DDS, MS, PhD, EBOS. Agregated Professor of Oral Surgery, Faculty of Medicine and Health Sciences. Master’s Degree Program in Oral Surgery and Implantology. University of Barcelona. Researcher of the IDIBELL institute, Barcelona (Spain); 3 MD, DDS, MS, PhD, EBOS, OMFS. Chairman and Professor of Oral and Maxillofacial Surgery, Faculty of Medicine and Health Sciences. University of Barcelona. Director of the Master’s Degree Program in Oral Surgery and Implantology (EHFRE International University/UCAM/FUCSO). Coordinator/Researcher of the IDIBELL Institute. Head of the Oral Surgery, Implantology and Maxillofacial Surgery Department of the Teknon Medical Center, Barcelona (Spain)

## Abstract

**Background:**

Oral bone regeneration techniques (OBRT) attempt to provide the appropriate bone volume and density to correctly accomplish dental implant treatments. The objective was to determine whether differences exist in the clinical outcomes of these techniques between diabetic and non-diabetic patients, considering the level of scientific evidence.

**Material and Methods:**

A systematic review following PRISMA statements was conducted in the PubMed, Scopus and Cochrane databases with the search terms: “Diabetes Mellitus”, “guided bone regeneration”, “bone regeneration”, “alveolar ridge augmentation”, “ridge augmentation”, bone graft*, “sinus floor augmentation”, “sinus floor elevation”, “sinus lift”, implant*. Articles were limited to those published less than 10 years ago and in English. Inclusion criteria were: human studies of all bone regeneration techniques, including at least 10 patients and the using OBRT in diabetic and non-diabetic patients. Non-human studies were excluded. They were stratified according to their level of scientific evidence related to SORT criteria (Strength of Recommendation Taxonomy).

**Results:**

The initial search provided 131 articles, after reading the abstracts a total of 33 relevant articles were selected to read the full text and analyzed to decide eligibility. Finally, seven of them accomplished the inclusion criteria: two controlled clinical trials, one cohort study and four case series.

**Conclusions:**

A low grade of evidence regarding the use of OBRT in diabetic patients was found. The recommendation for this intervention in diabetic patients is considered type C due to the high heterogeneity of the type of diabetic patients included and the variability of the techniques applied.

** Key words:**Diabetes Mellitus, guided bone regeneration, bone regeneration.

## Introduction

Alveolar crest volume reduction is a common consequence of tooth loss and can be, not only a difficulty for the treatment planning, but also a contraindication for dental implants placement. For this reason, bone augmentation procedures may be required before implant therapy in areas with moderate to severe bone loss. Bone regeneration techniques, include alveolar bone augmentation (ABA), guided bone regeneration (GBR) and sinus lift (SL) procedures.

Systemic diseases including diabetes mellitus (DM) could be formally considered a contraindication (1–3) for these interventions, especially in case of associated implant installation, due to its vascular and immune deficiencies.

One of the most prevalent systemic diseases worldwide is diabetes mellitus (DM), a chronic metabolic disorder composed of two subtypes: Type 1 DM involves 5-10% of diabetic patients and is an autoimmune disorder related to the destruction of pancreatic β-cells and the consequent deficit in insulin production; Type 2 DM, involves 90-95% of diabetic patients and is a multifactorial disease caused by environmental factors (e.g. obesity and sedentary lifestyle, corticosteroids intake) which lead to peripheral or cellular insulin resistance in genetically predisposed cases (4). The final result of decreased pancreatic production (type 1 DM) or peripheral cellular insensibility to insulin (type 2 DM) is an increase in blood glucose levels (hyperglycemia).

Treatment in both types of DM is focused on achieving a proper glycemic control in order to prevent the development of medical complications (3,5). In the long term, hyperglycemia promotes vascular complications which are one of the main causes of morbidity and mortality in this type of patients (6).

Chronic hyperglycemia also affects different tissue structures and produces an inflammatory effect, which results in a negative imbalance in the process of bone remodelation due to a decrease in bone formation rather than an increase in reabsorption as a consequence of the inhibitory effect of hyperglycemia on osteoblastic differentiation, impairment of parathyroid hormone activity which regulates phosphorus and calcium metabolisms (7) and a reduction in adherence, growth and accumulation of the extracellular matrix, as it has been demonstrated in experimental models that mineral homeostasis and osteoid production are significantly decreased in DM patients (6). Conversely, such models also showed that a persistent normoglycemic levels is directly correlated with an increased bone matrix and osteoid generation at a rate similar to controls, increasing bone formation around the dental implants (6-9). DM patients with good glycemic control also demonstrate a markedly reduced rate of periodontal bone loss, and a lower incidence of postoperative complications, compared to those with an inadequate one (3). Several studies have reported that the former group show successful dental implant rates similar to non-DM patients (4,11).

The main objective of this work was to review the current literature, taking into account the level of scientific evidence, to ascertain the rate of success of the oral bone regeneration techniques (OBRT) in DM patients.

## Material and Methods

In October 2017 an electronic search was performed using PubMed, Scopus and Cochrane databases, following the PRISMA statements (10), in order to answer the following PICO question: “In patients with DM compared with non-diabetics are there the same results of OBRT in terms of bone regeneration, complications and success of dental implants?”

The search was limited to articles published in English between 2007 and 2017 with the search terms: “diabetes mellitus”, “bone regeneration”, “guided bone regeneration”, “alveolar ridge augmentation”, “bone graft*”, “sinus floor elevation” and “implant*”. A second search was carried out employing Boolean operators such as “OR/AND” and synonyms of the keywords to obtain articles that included two or more of the terms.

Finally, the Mesh Terms of the keywords were looked for, and a final search was performed: ((“Diabetes Mellitus”[Mesh]) AND (“guided bone regeneration” OR “bone regeneration” OR “alveolar ridge augmentation” OR “ridge augmentation” OR bone graft* OR “sinus floor augmentation” OR “sinus floor elevation” OR “sinus lift”)) AND implant*.

Inclusion criteria were: human studies, published in English from 2007 to 2017, regarding the use of OBRT in DM and non-DM patients. Exclusion criteria were non-human studies and case series including less than 10 patients or case-control studies.

Articles were then stratified according to the level of scientific evidence using SORT criteria (11). On the basis of their scientific quality a grade of recommendation was given respect to the use OBRT in DM patients.

## Results

The final electronic search performed on 31st October 2017 provided 58 articles from Pubmed, 72 from Scopus and 7 from Cochrane databases. Another three articles were added after an additional hand search based on the references of the papers already found.

After removing duplicates 131 articles were obtained, and after reading the abstracts, a total of 33 relevant articles were identified and selected to read the full text. Finally seven articles fulfilling inclusion criteria were included (1,5,12-16). The remaining 26 were excluded due to being descriptive studies (14,15), animal studies (16-24), no-DM patients (25), not performing bone regeneration in the oral region (26) and not specifying whether OBRT was employed in DM patients (27–30).

**Figure 1:**
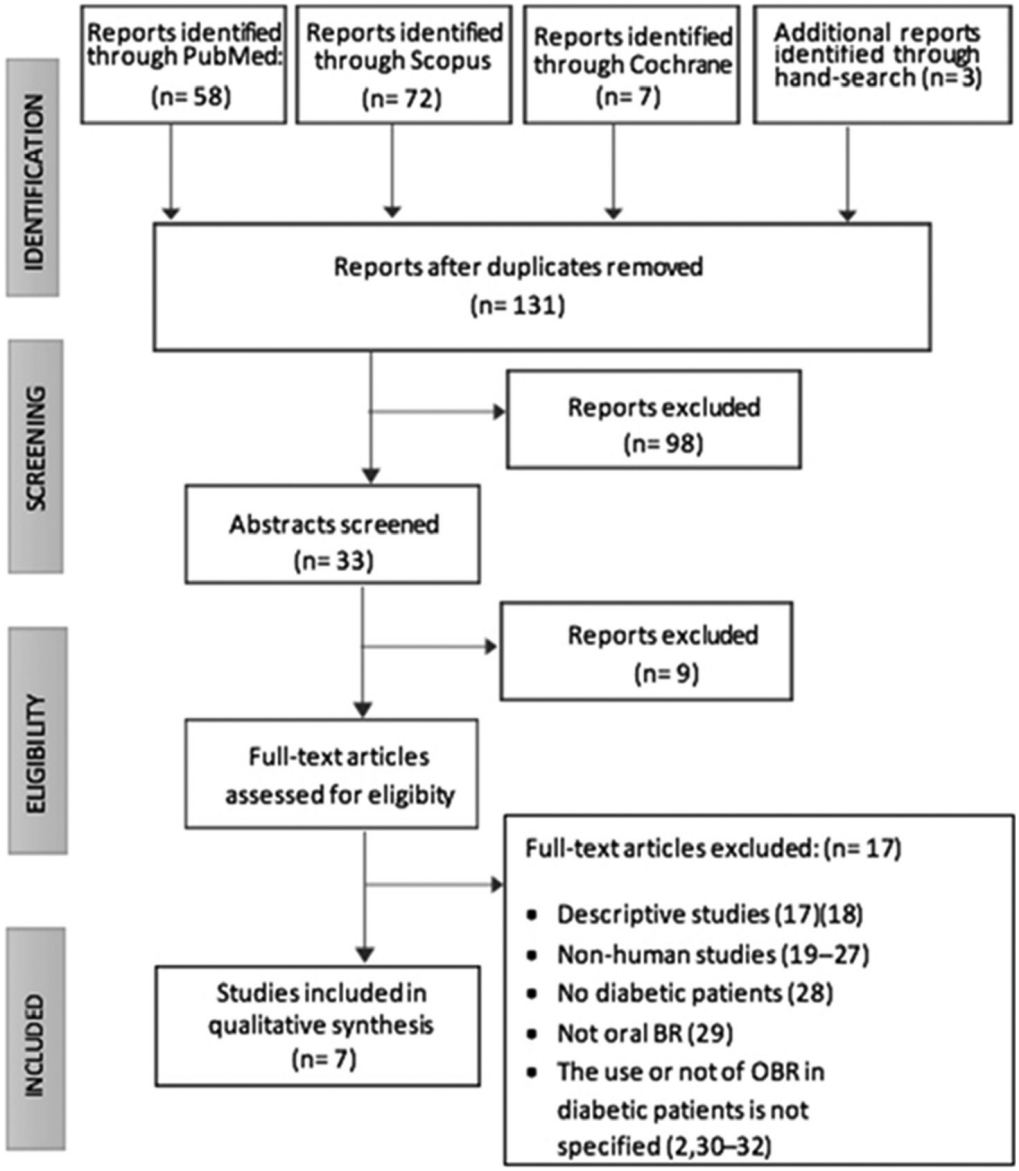
Flow-chart of the review process modified from PRISMA statement (10).

The flow-chart of the review process modified from the PRISMA statement (10) is shown in Figure 1. Two out of the seven selected articles were controlled clinical trials (1,5), one a retrospective cohort study (12) and four were retrospective case series (13-16).

The selected articles were classified according to the level of scientific evidence following SORT criteria (11): 3 of scientific evidence level 2 (1,5,12), and the remaining ones (13–16) of level 3.  Table 1 summarizes the level of evidence of each study and the reasons for classifications.

**Table 1:**
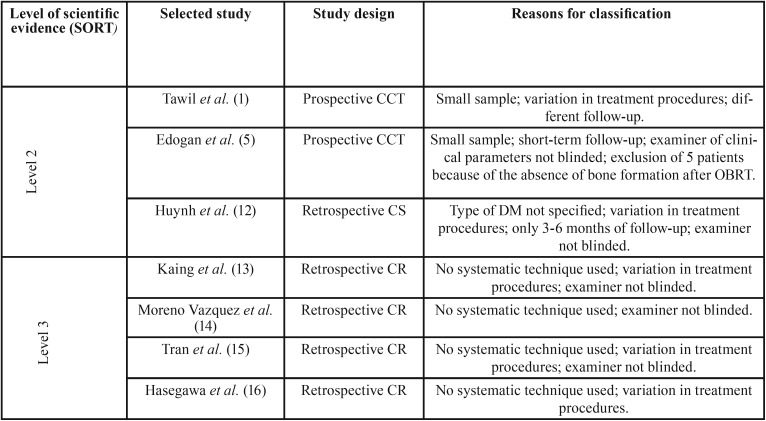
Studies level of classification and the reasons for classification. CCT: controlled clinical trials, CR: case reports, CS: cohort study.

Study design, duration, number of patients, gender, mean age, DM type, and OBRT applied are shown in  Table 2.

**Table 2:**
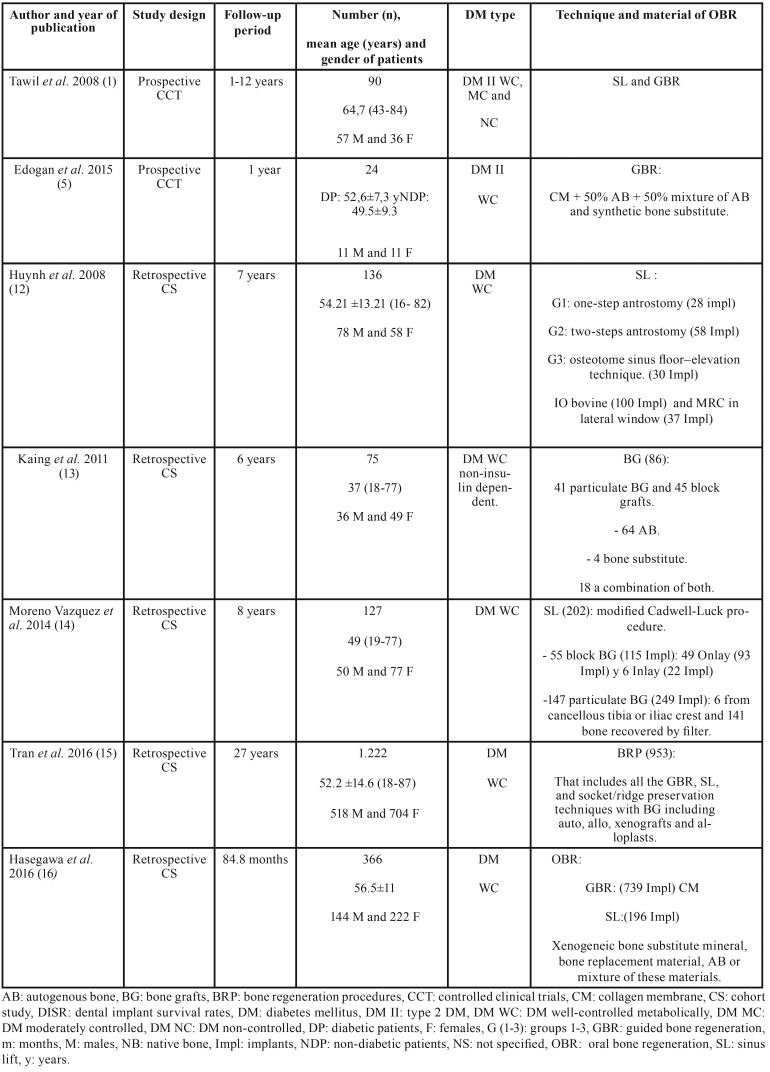
Study design, duration, number of patients, gender, mean age, DM type, and OBRT applied.

The main characteristics of each study: number of patients who underwent OBRT (DM patients and non-DM ones), number of implants placed in native and regenerated bone, evaluation technique, failure criteria, and results are synthetized in  Table 3 and  Table 4.

**Table 3:**
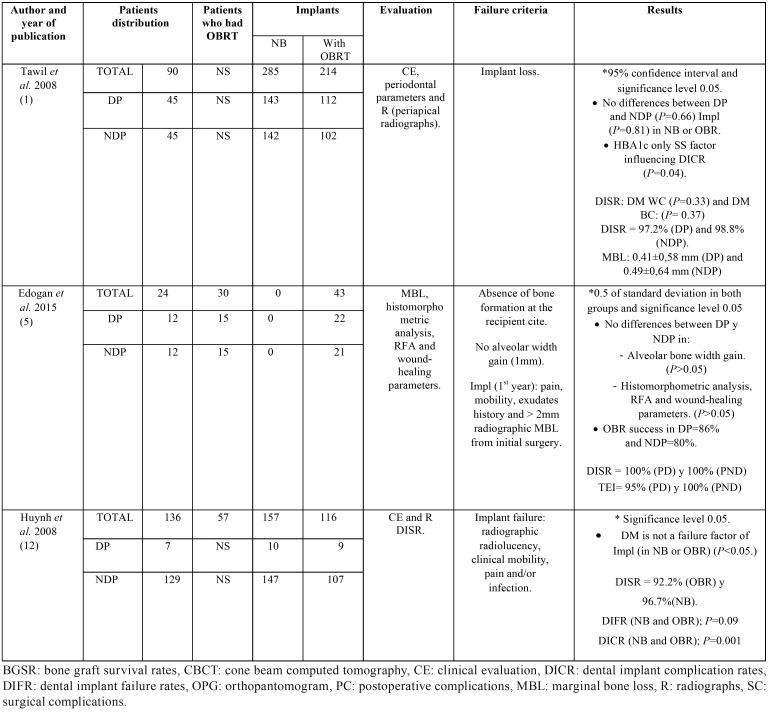
Number of patients who underwent OBRT (DM patients and non-DM ones), number of implants placed in native and regenerated bone, evaluation technique, failure criteria, and results.

**Table 4:**
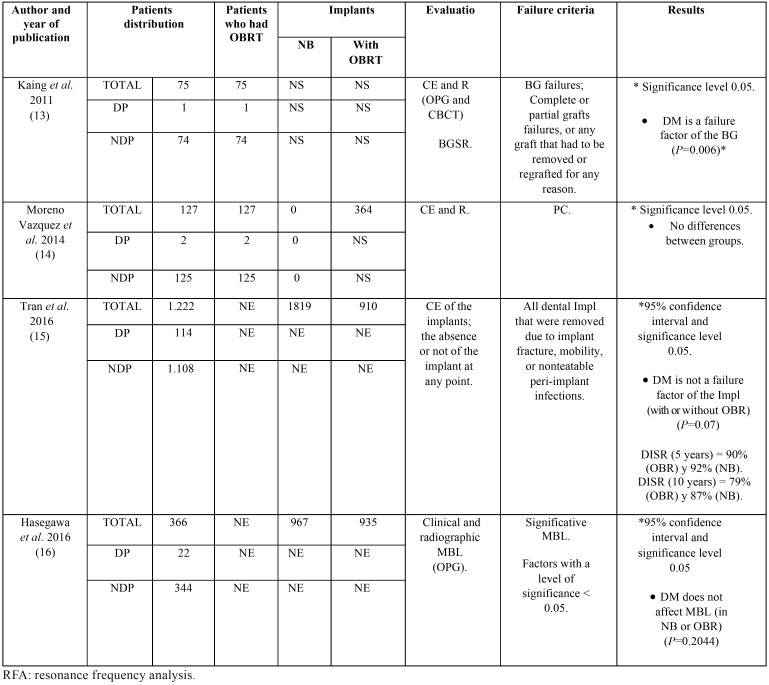
Continuation of table 3.

Regarding OBRT in DM patients the strength of recommendation, on the basis of the level of evidence in the available data, is level C.

Because the inconsistency of the patients’ characteristics, the methodology used and parameters of comparison, it does not allow to carry on a methanalysis which could provide quantitative conclusions about the benefits of OBRT in DM patients. Due to the high heterogeneity of the included studies it is not possible to perform a meta-analysis of the data.

## Discussion

Diabetic patients have an increased incidence of periodontal disease and bone reabsorption (5), greater loss of alveolar bone (33,34) and more post-operative complications following implant placement surgery than non-DM ones (6).

Due to this increased incidence of risk factors and complications, it has been shown that the success rate of dental implants in DM patients should be lower than in non-DM population (1,5,6,19), nevertheless, a good metabolic control can improve the survival rate of dental implants in such patients (1,3,6,20,21). Well-controlled DM is therefore not considered a full contraindication for implant therapy even though current literature suggests a certain decrease in the success rate of concurrent surgical procedures as would be the case of OBRT (5).

In our review process it has been identified some studies evaluating the success of bone regeneration techniques in DM animals (22–30), however, there were few regarding DM in humans despite the safety of performing implant therapy in such condition having been proven. Studies in non-controlled DM patients are even scarcer, probably due to the previously mentioned complications and ethical reasons. Only one publication with respect to OBR in non-controlled DM patients (HbA1c > 9%) was identified and includes only one DM patient underwent surgery (1).

The regeneration techniques applied in the seven selected studies for systematic review analysis include GBR, SL, and bone grafts. Out of the 7, only one (5) aimed at comparing OBRT clinical results in DM and non-DM patients. The objective of the remaining six was to identify the predictive failure factors of certain OBRTs (13,14) and for dental implants (1,12,16), as well to compare dental implant survival rate between native and regenerated bone (15).

Some of these studies had a limited number of DM patients and a marked disproportion between both groups (DM and non-DM) (12–14). In addition, relevant DM patient data were only specified in two articles regarding illness duration, treatment applied to control the metabolic state, and pre/post-surgery HbA1c levels (1,5). Only Tawil *et al.* (1) considered the different glycemic levels as a possible individual risk factor for implant therapy and OBRT, concluding that HbA1c levels were the only multivariable and independent factor affecting the rate of complication.

On the other hand, as the principal aim of OBRT is to permit posterior implant therapy to be correctly performed, it is difficult to clearly separate the implant success/failure or complication rates from each other (15,16), neither to compare OBRT because there is no unified methodology to evaluate the results. One study only specified the OBR procedures without detailing the implants placed later, as Kaaing *et al.* (13), or only described the implants placed in regenerated bone, ignoring the patients who underwent OBRT or the number of OBR procedures performed, as Tran *et al.* (15), Hasegawa *et al.* (16), and Tawil *et al.* (1). Huynh *et al.* (12) report the number of patients who underwent OBRT, but do not specify whether they were DM or non-DM ones.

Tran *et al.* (15) and Hasegawa *et al.* (16) counted the number of implants placed in native and regenerated bone and compared survival rates, without specifying the number of implants or techniques in both groups of patients. Huynh *et al.* (12) also calculated implant survival and success rates, specifying how many of each type were placed in DP and non-DP patients, and the total number who underwent OBRT. In the studies by Erdogan *et al.* (5) and Moreno Vazquez *et al.* (14) all the patients underwent OBRT and subsequent implant installation. Erdogan *et al.* (5) provide exact information regarding the number of placed implants (all with OBRT) in DM and non-DM patients, while Moreno Vazquez *et al.* (14) do not specify the number of implants placed in each group. In the case of Kaing *et al.* (13), all the patients had an OBRT and the study is focused on their results, without specifying the number of implants later inserted. Finally, in the study by Tawil *et al.* (1), patients were divided into DM and non-DM, and in both groups implants were placed with and without OBRT. In this way, they could estimate the differences related to the results between implants installed in regenerated bone and conventional ones in the two types of patients.

Finally, other factors contributing to the inconsistency of the results were: not mentioning or not classifying the bone defect, diagnostic methodology, and the criteria and methodology employed to evaluate the OBRT success or failure. Nonetheless, one study (13) did report differences in OBRT results in DM patients compared to non-DM ones.

## Conclusions

As OBRT is a fairly new surgical intervention, and the available literature is not only scarce but extremely heterogeneous, it is not possible to categorically assert the reliability of this procedure in well-controlled DM patients. As consequence, and following the principles of evidence-based odontology, the present analysis reports a grade C of recommendation regarding its use.

## References

[1] Tawil G, Younan R, Azar P, Sleilati G (2008). Conventional and advanced implant treatment in the type II diabetic patient: surgical protocol and long-term clinical results. Int J Oral Maxillofac Implants.

[2] Omran MT, Miley DD, McLeod DE, Garcia MN (2015). Retrospective assessment of survival rate for short endosseous dental implants. Implant Dent.

[3] Javed F, Romanos GE (2009). Impact of diabetes mellitus and glycemic control on the osseointegration of dental implants: A systematic literature review. J Periodontol.

[4] Erdogan Ö, Charudilaka S, Tatli U, Damlar I (2010). A review on alveolar bone augmentation and dental implant success in diabetic patients. Oral Surg.

[5] Erdogan Ö, Uçar Y, Tatlı U, Sert M, Benlidayı ME, Evlice B (2015). A clinical prospective study on alveolar bone augmentation and dental implant success in patients with type 2 diabetes. Clin Oral Implant Res.

[6] Mellado-Valero A, Ferrer García JC, Herrera Ballester A, Labaig Rueda C (2007). Effects of diabetes on the osseointegration of dental implants. Med Oral Patol Oral Cir Bucal.

[7] Locatto ME, Abranzon H, Caferra D, Fernandez MC, Alloatti R, Puche RC (1993). Growth and development of bone mass in untreated alloxan diabetic rats. Effects of collagen glycosylation and parathyroid activity on bone turnover. Bone Miner.

[8] Casap N, Nimri S, Ziv E, Sela J, Samuni Y (2008). Type 2 diabetes has minimal effect on osseointegration of titanium implants in Psammomys obesus. Clin Oral Implant Res.

[9] Loe H (1993). Periodontal disease. The sixth complication of diabetes mellitus. Diabetes Care.

[10] Moher D, Liberati A, Tetzlaff J, Altman DG (2010). Preferred reporting items for systematic reviews and meta-analyses: The PRISMA statement. Int J Surg.

[11] Ebell MH, Siwek J, Weiss BD, Woolf SH, Susman J, Ewigman B (2004). Strength of Recommendation Taxonomy (SORT): A Patient-Centered Approach to Grading Evidence in the Medical Literature. J Am Board Fam Pract.

[12] Huynh-Ba G, Friedberg JR, Vogiatzi D, Ioannidou E (2008). Implant failure predictors in the posterior maxilla: a retrospective study of 273 consecutive implants. J Periodontol.

[13] Kaing L, Grubor D, Chandu A (2011). Assessment of bone grafts placed within an oral and maxillofacial training programme for implant rehabilitation. Aust Dent J.

[14] Moreno Vazquez JC, Gonzalez de Rivera AS, Gil HS, Mifsut RS (2014). Complication Rate in 200 Consecutive Sinus Lift Procedures : Guidelines for Prevention and Treatment. J Oral Maxillofac Surg.

[15] Tran DT, Gay IC, Diaz-Rodriguez MSJ, Parthasarathy K, Weltman MSR, Friedman MSL (2016). Survival of Dental Implants Placed in Grafted and Nongrafted Bone: A Retrospective Study in a University Setting Duong. Int J Oral Maxillofac Implant.

[16] Hasegawa M, Hotta Y, Hoshino T, Ito K, Komatsu S, Saito T (2016). Long-term radiographic evaluation of risk factors related to implant treatment : suggestion for alternative statistical analysis of marginal bone loss. Clin Oral Implant Res.

[17] Javed F, Näsström K, Benchimol D, Altamash M, Klinge B, Engström P (2007). Comparison of periodontal and socioeconomic status between subjects with type 2 diabetes mellitus and non-diabetic controls. J Periodontol.

[18] Taylor GW, Burt BA, Becker MP, Genco RJ, Shlossman M, Knowler WC (1998). Non-insulin dependent diabetes mellitus and alveolar bone loss progression over 2 years. J Periodontol.

[19] de Molon RS, Morais-Camilo JA, Verzola MH, Faeda RS, Pepato MT, Marcantonio E Jr (2013). Impact of diabetes mellitus and metabolic control on bone healing around osseointegrated implants : removal torque and histomorphometric analysis in rats. Clin Oral Implants Res.

[20] Morris HF, Ochi S, Winkler S (2000). Implant survival in patients with type 2 diabetes: placement to 36 months. Ann Periodontol.

[21] Farzad P, Andersson L, Nyberg J (2002). Dental implant treatment in diabetic patients. Implant Dent.

[22] Camargo WA, de Vries R, van Luijk J, Hoekstra JW, Bronkhorst EM, Jansen JA (2017). Diabetes mellitus and bone regeneration: A systematic review and meta-analysis of animal studies. Tissue Eng Part B Rev.

[23] Hamann C, Goettsch C, Mettelsiefen J, Henkenjohann V, Rauner M, Hempel U (2011). Delayed bone regeneration and low bone mass in a rat model of insulin-resistant type 2 diabetes mellitus is due to impaired osteoblast function. Am J Physiol Endocrinol Metab.

[24] Retzepi M, Lewis MP, Donos N (2010). Effect of diabetes and metabolic control on de novo bone formation following guided bone regeneration. Clin Oral Implant Res.

[25] Retzepi M, Calciolari E, Wall I, Lewis MP, Donos N (2018). The effect of experimental diabetes and glycaemic control on guided bone regeneration: histology and gene expression analyses. Clin Oral Implant Res.

[26] Jardini MA, Tera TM, Meyer AA, Moretto CM, do Prado RF, Santamaria MP (2016). Guided bone regeneration with or without a collagen membrane in rats with induced diabetes mellitus: Histomorphometric and immunolocalization analysis of angiogenesis and bone turnover markers. Int J Oral Maxillofac Implant.

[27] Gomes MF, Destro MF, Banzi EC, Vieira EM, Morosolli AR, Goulard Md (2008). Optical density of bone repair after implantation of homogenous demineralized dentin matrix in diabetic rabbits. Braz Oral Res.

[28] Li H, Liao H, Bao C, Xiao Y, Wang Q (2017). Preparation and Evaluations of Mangiferin-Loaded PLGA Scaffolds for Alveolar Bone Repair Treatment Under the Diabetic Condition. AAPS PharmSciTech.

[29] Timóteo CA, Aranega AM, Shinohara EH, Coléte JZ (2017). Bone repair process in defects of diabetic rats filled with autogenous bone graft and covered with homogenous bone matrix membrane or polytetrafluoroethylene membrane. Int J Oral Maxillofac Implants.

[30] Lee SB, Retzepi M, Petrie A, Hakimi AR, Schwarz F, Donos N (2012). The effect of diabetes on bone formation following application of the GBR principle with the use of titanium domes. Clin Oral Implant Res.

